# Comprehensive Quantification of Collagen, Elastin, and Glycosaminoglycans in the Human Facial Dermis: Insights From a Pilot Study

**DOI:** 10.7759/cureus.81419

**Published:** 2025-03-29

**Authors:** Iman Bouchelkia, Chakravarthy Sadacharan

**Affiliations:** 1 Biomedical Sciences, Tilman J. Fertitta Family College of Medicine, Houston, USA; 2 Anatomy, Tilman J. Fertitta Family College of Medicine, Houston, USA

**Keywords:** collagen-elastin matrix, dermal extracellular matrix, dermatology, glycosaminoglycans, skin repair

## Abstract

Background

Age-related alterations in the dermal extracellular matrix (ECM) drive skin aging and are linked to numerous dermatological conditions, including diminished wound healing and compromised skin integrity. Our study aims to quantify and compare the distribution of collagen, elastic fibers, and glycosaminoglycans (GAGs) across different ages and analyze their patterns in vertical sections of the facial dermis.

Methods

Skin samples were collected from the under-eye (UE) and forehead (FH) regions of 24 male and female human cadavers aged 59-87. Samples were processed histologically. Collagen, elastic fibers, and GAG content were stained using trichrome, van Gieson, and alcian blue stains, respectively. The data was collected using ImageJ software. Two-way ANOVA with post hoc Tukey’s test was used to analyze differences across regions and stain types.

Results

The FH region showed significantly higher GAGs in horizontal sections compared to vertical (p < 0.0001), with no significant differences in collagen (p = 0.9968) or elastic fibers (p = 0.8762). In the UE region, GAGs were also higher in horizontal sections (p = 0.0001), while collagen (p = 0.3954) and elastic fibers (p > 0.9999) showed no differences. Comparing FH and UE, vertical sections showed significant differences in collagen (p = 0.021) and GAGs (p = 0.0027), with higher values in FH. Horizontal sections showed significantly higher GAGs in FH (p = 0.001), with no differences in collagen (p = 0.5709) or elastic fibers (p > 0.9999). ECM changes were significant with age, but no differences were observed based on gender.

Conclusions

These findings offer novel insights into the structural and functional differences in the facial dermis, which can inform targeted anti-aging treatments, improve strategies for wound healing, and guide personalized dermatological interventions for skin integrity and rejuvenation.

## Introduction

The skin’s ability to maintain its structural integrity, elasticity, and resilience declines with age, resulting in delayed wound healing, reduced scar quality, and increased susceptibility to dermatologic conditions. These changes are primarily driven by alterations in the dermal extracellular matrix (ECM). ECMs are complex, well-organized three-dimensional networks that play vital roles in providing structural support, facilitating tissue organization and remodeling, and regulating cellular processes [1]. The key constituents of the ECM include collagen, proteoglycans, glycosaminoglycans (GAGs), elastin and elastic fibers, and other proteins/glycoproteins [1]. Each component of the ECM contributes uniquely to its functions. Dermal collagen represents the most abundant ECM protein, constituting 90% of the dry weight of the skin [2]. Its role is to maintain the structural integrity and elasticity of the skin. The papillary layer has a dense mesh structure consisting of randomly arranged thin collagen fibers (mainly type III) and immature elastic fibers, while the reticular layer consists of fibrous and highly ordered thick bundles of collagen fibers (mainly type I) [3]. Elastin is a critical skin protein that combines with microfibrils to form elastic fibers that provide stretch and recoil to the skin [4]. It has a slow turnover rate, making it vulnerable to damage from various factors. GAGs make up 0.1-0.3% of total skin weight, with six- to seven-fold more GAG present in the dermis compared with the epidermis [5].

Over time, the ECM undergoes structural alterations due to skin aging, leading to a reduction in both the quantity and length of collagen fibers. It has been shown that the overall collagen content of the skin surface is known to decline by approximately 1% per year, weakening the dermis’ flexibility and causing wrinkles [6]. Aging leads to the degradation and reorganization of collagen, elastin, and GAGs, contributing to wrinkles, decreased wound healing capacity, and skin thinning. The earliest signs of facial aging are often visible in the periorbital area, with changes in skin color and appearance [7]. The under-eye (UE) region is particularly prone to volume loss, presenting as tear trough formation, fat atrophy, and fat descent [8,9]. In contrast, the forehead (FH) region begins to flatten and form wrinkles due to the contraction of the frontalis muscle. Loss of dermal thickness and hydration in the FH contributes to skin flattening.

The directionality of fibers may influence tissue repair and surgical outcomes, which is why understanding vertical vs. horizontal sections is important. This contributes to the skin’s biomechanical properties, such as how the skin responds to stretching, shearing, and mechanical stress. The skin’s collagen and elastic fibers have anisotropic properties, which have important implications for surgical incision planning and wound healing. Incisions made parallel to Langer’s lines, also known as relaxed skin tension lines, heal with less scarring compared to those made perpendicular to the lines [10]. Despite this, limited data exist on how collagen, elastin, and GAGs vary by age and fiber orientation, particularly in the vertical and horizontal sections of facial dermal skin. While prior studies have examined the correlation between elastin and collagen fibers in body skin [11], facial ECM composition remains underexplored. Given the unique biomechanical demands of the FH and UE region, a better understanding of ECM orientation could inform surgical and dermatological practices.

Thus, the purpose of this study was to quantify and compare the distribution of collagen, elastic fibers, and GAGs in the UE and FH regions across different ages and genders in both vertical and horizontal sections. By addressing this gap, our findings provide new insights into facial ECM remodeling with age and highlight potential target areas for dermatological and aesthetic interventions.

## Materials and methods

A total of 92 skin samples were collected from the UE and FH regions (medial sides) of human donors aged 59-87 years. Samples were obtained in both horizontal and vertical orientations to assess variations in collagen, elastic fibers, and GAGs. Prior to collection, the gross appearance of the skin was examined to ensure healthy tissue quality. Donors showing clinical or histopathological indicators of advanced cutaneous pathology were excluded from the study. The sample set included both male and female donors, allowing for gender-based comparisons of ECM composition. The specimens were ethically sourced from accredited donation programs that adhere to consent and legal guidelines and, as cadaveric tissue, are exempt from institutional review board oversight as they do not fall under the classification of human subject research.

Tissue processing and staining

All samples were processed at the University of Houston’s Center for Nuclear Receptors and Cell Signaling. Skin tissues were embedded and processed using standard histological staining protocols. The following stains were used to identify collagen, elastic fibers, and GAGs: alcian blue to visualize GAG content, van Gieson to highlight elastic fibers, and trichrome stain to identify collagen fibers.

Tissue samples were fixed in 10% neutral buffered formalin (Sigma-Aldrich, St. Louis, Missouri, United States) or Bouin’s solution (Polysciences, Warrington, Pennsylvania, United States), processed through graded ethanol, embedded in paraffin, and sectioned at 4-5 µm using a Leica RM2235 microtome (Leica Biosystems, Nussloch, Germany). Sections were then deparaffinized and rehydrated before staining.

For alcian blue staining, sections were incubated with 1% alcian blue (pH 2.5 or 1.0) for 30 minutes, washed in distilled water, and counterstained with Nuclear Fast Red (Thermo Fisher Scientific, Waltham, Massachusetts, United States) or hematoxylin (Sigma-Aldrich). After dehydration in graded ethanol and xylene, slides were mounted with a Permount Mounting Medium (Fisher Scientific, Pittsburgh, Pennsylvania, United States).

For Masson’s trichrome staining, sections were stained with Weigert’s Hematoxylin (five minutes), Biebrich Scarlet-Acid Fuchsin (five minutes), and differentiated with phosphotungstic/phosphomolybdic acid (five minutes) (Electron Microscopy Sciences, Hatfield, Pennsylvania, United States). This was followed by aniline blue staining (5 min) and fixation with 1% acetic acid (one minute). After dehydration and clearing in xylene, sections were mounted. Collagen fibers stained blue, muscle and cytoplasm red, and nuclei black or dark blue.

For van Gieson staining, sections were incubated with Weigert’s Hematoxylin (five minutes), followed by van Gieson solution (acid fuchsin + picric acid, two to three minutes). After washing and dehydration, slides were mounted with Cytoseal XYL (Thermo Fisher Scientific). Collagen fibers stained red, muscle and cytoplasm yellow, and nuclei black or blue-black, allowing differentiation of connective tissue components.

Image and statistical analysis

Stained tissue sections were photographed using a Nikon Ti2 inverted microscope (Nikon Instruments, Inc., Konan, Japan) under consistent magnification and lighting conditions. High-resolution images were captured in TIFF format to preserve detail.

Image analysis was performed using Fiji (ImageJ 2.0, NIH, USA) to quantify collagen, elastic fibers, and GAG content. Raw images were converted to 8-bit grayscale, and a color thresholding algorithm was applied to isolate stained regions while excluding background noise. The Analyze Particles function quantified stained areas, with pixel measurements converted to surface area units (µm²) based on a calibrated scale bar. The proportion of stained regions was calculated by measuring the pixel count corresponding to the targeted stain, with values converted to a percentage representing the relative stained area. Data were normalized to ensure accurate quantification of histological features.

Statistical analysis was performed using GraphPad Prism. A two-way ANOVA was conducted to assess differences across regions (FH vs. UE) and stain types (collagen, elastic fibers, and GAGs). Post hoc Tukey’s test was used to further explore significant differences between groups, with adjusted p-values calculated to determine statistical significance.

## Results

Orientation comparison within the UE and FH region

**Figure 1:**
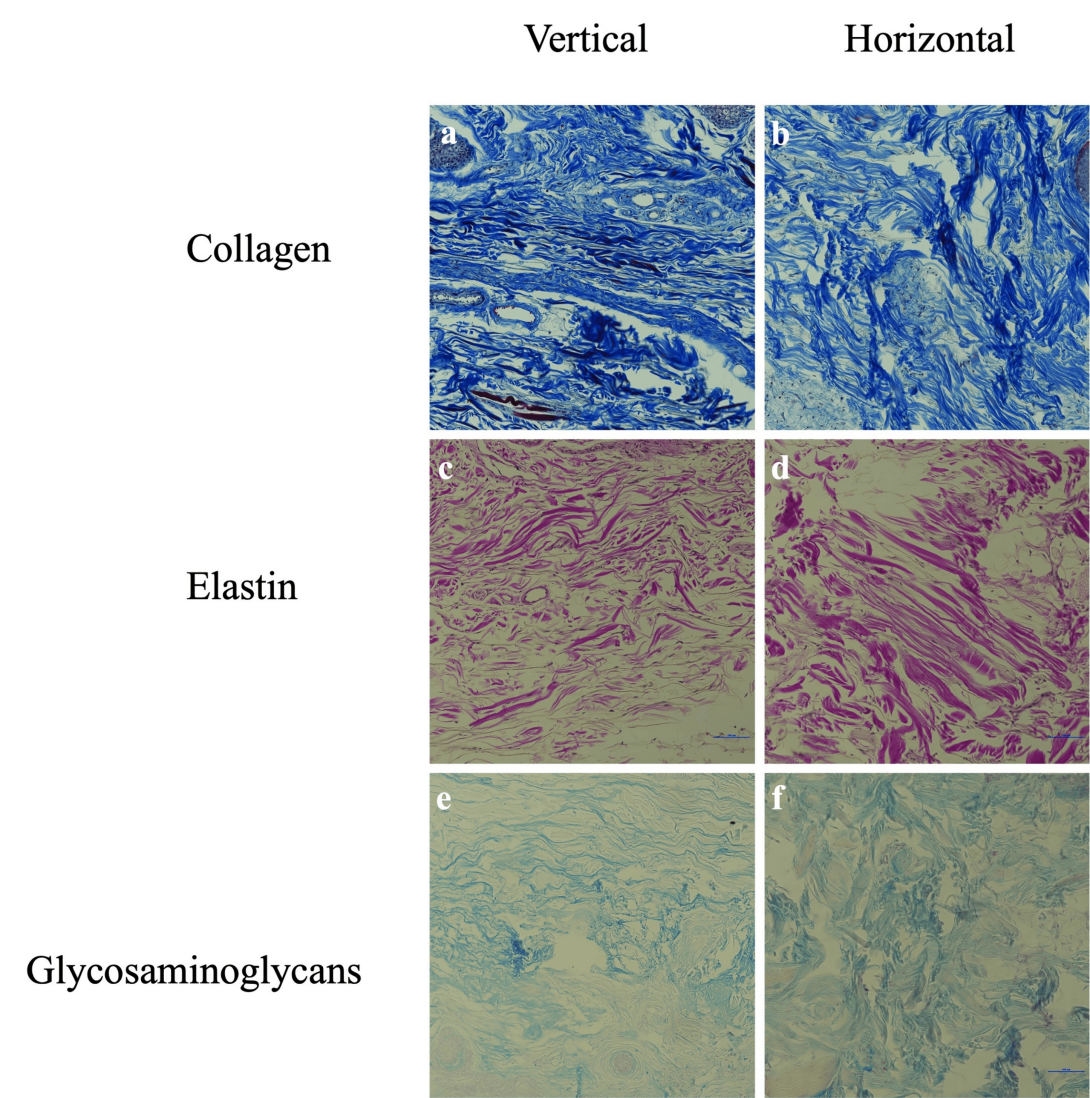
Histological comparison of collagen, elastin, and GAGs in vertical and horizontal orientations of FH skin tissue at 20x magnification FH, forehead; GAG, glycosaminoglycan

For elastic fibers in the FH, no statistically significant difference was observed between horizontal and vertical sections. However, for GAGs, the horizontal and vertical orientations differed significantly. Collagen fibers showed no significant difference. Histological analysis of the tissue samples supports these findings, with comparable staining patterns for collagen and elastin across orientations, while GAG staining exhibits a marked increase in intensity in the horizontal sections (Figure 1).

The mean quantitative fractions with SDs for elastic fibers, GAGs, and collagen fibers in horizontal and vertical orientations, as well as the range of values, mean differences between the orientations, and adjusted p-values of the FH skin, are summarized in Table 1.

**Table 1:** Descriptive statistics of elastic fibers, GAGs, and collagen fibers between horizontal and vertical orientations in the FH measured in square micrometers FH, forehead; GAG, glycosaminoglycan

Component	Horizontal			Vertical			Mean difference	Adjusted p-Value
Descriptive statistics	Mean	Range	SD	Mean	Range	SD	Statistical comparison	Statistical comparison
Elastic fibers	0.000201	0.000538	0.000155	0.000507	0.000562	0.000159	0.000306	0.8762 (ns)
GAGs	0.001954	0.003521	0.00095	0.001141	0.002729	0.000852	-0.00081	<0.0001 (****)
Collagen fibers	0.002198	0.003397	0.000895	0.001988	0.001512	0.000449	-0.00021	0.9968 (ns)

**Figure 2:**
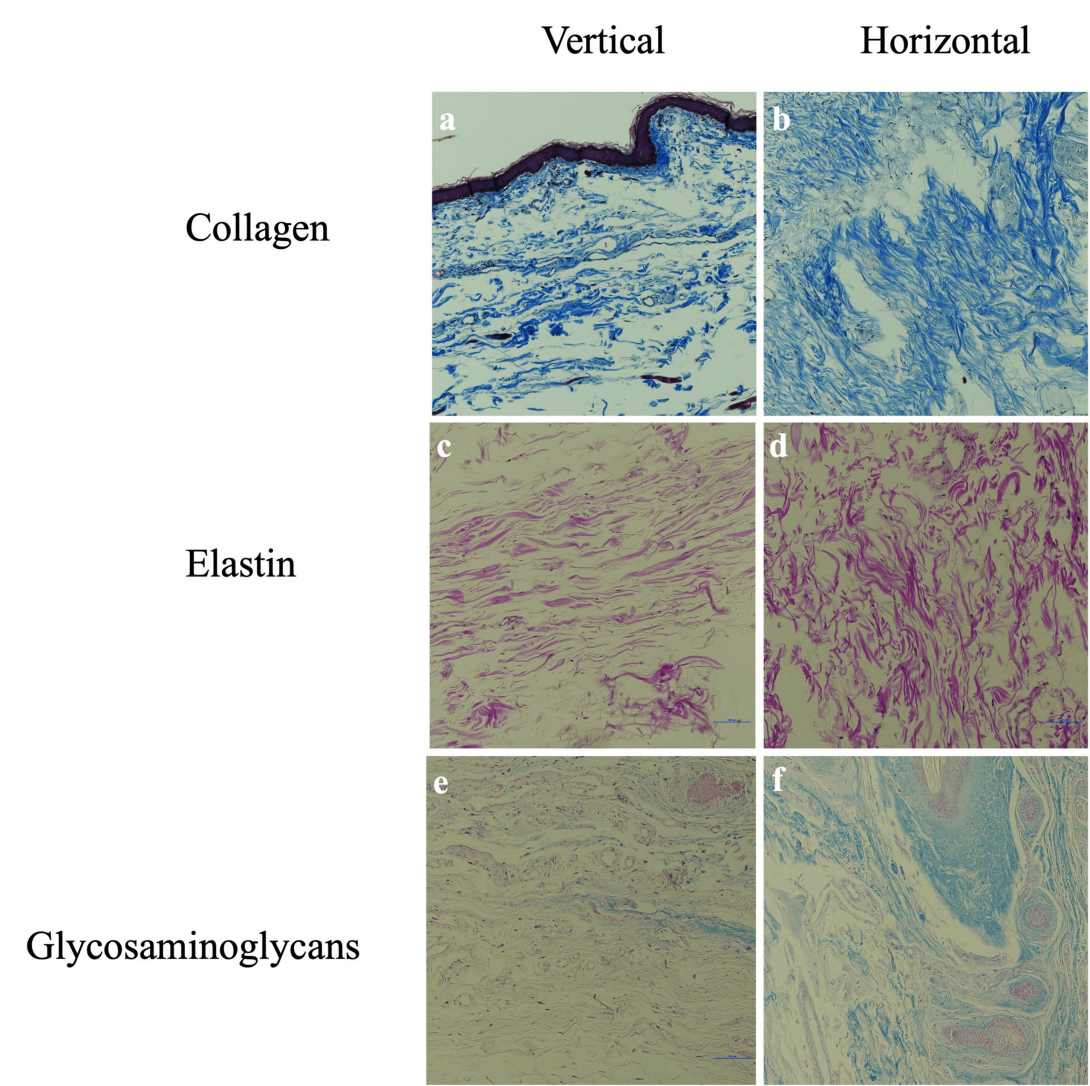
Histological comparison of collagen, elastin, and GAGs in vertical and horizontal orientations of undereye skin tissue at 20x magnification GAG, glycosaminoglycan

In the UE region, elastic fibers showed no significant difference between horizontal and vertical orientations. GAG content differed significantly between orientations, while collagen fibers showed no significant difference. Histological evaluation of the UE tissue samples reveals similar staining patterns for collagen and elastin fibers regardless of orientation, while GAG staining shows noticeably greater intensity in the horizontal sections (Figure 2).

The mean quantitative fractions with SDs for elastic fibers, GAGs, and collagen fibers in horizontal and vertical orientations, as well as the range of values, mean differences between the orientations, and adjusted p-values of the UE skin, are summarized in Table 2.

**Table 2:** Descriptive statistics of elastic fibers, GAGs, and collagen fibers between horizontal and vertical orientations UE measured in square micrometers GAG, glycosaminoglycan; UE, under-eye

Component	Horizontal			Vertical			Mean difference	Adjusted p-Value
Descriptive statistics	Mean	Range	SD	Mean	Range	SD	Statistical comparison	Statistical comparison
Elastic fibers	0.000203	0.000339	9.23E-05	0.00019	0.00039	0.000134	-1.28E-05	>0.9999 (ns)
GAGs	0.00125	0.000956	0.000234	0.000475	0.000501	0.000147	-0.00077	0.0001 (***)
Collagen fibers	0.00182	0.002497	0.000853	0.001405	0.003623	0.001078	-0.00041	0.3954 (ns)

Regional comparisons of the FH and UE

For vertical sections, elastic fibers did not show a statistically significant difference between the FH and UE regions. GAGs demonstrated a significant difference, as did collagen fibers. In horizontal sections, elastic fibers showed no significant difference between the FH and UE regions. GAGs differed significantly, while collagen fibers showed no significant difference (Table 3).

**Table 3:** Descriptive statistics for regional comparisons of elastic fibers, GAGs, and collagen fibers in vertical and horizontal sections of the FH and UE regions measured in square micrometers FH, forehead; GAG, glycosaminoglycan; UE, under-eye

Section orientation	Collagen		Elastin		GAGs	
Statistical comparison	Mean difference	p-Value	Mean difference	p-Value	Mean difference	p-Value
Horizontal	0.000378	0.5709 (ns)	-2E-06	>0.9999 (ns)	0.000704	0.001 (***)
Vertical	0.000583	0.021 (*)	0.000317	0.8417 (ns)	0.000666	0.0027 (**)

Aging and gender differences in ECM

Significant changes were observed in the ECM with age, including a decline in overall ECM components, indicating reduced skin hydration and elasticity (p = 0.0001). No significant differences in ECM composition were observed between male and female cadaveric skin samples (p = 0.7759), suggesting that ECM alterations are predominantly influenced by aging rather than gender.

## Discussion

Skin aging is a multifactorial process influenced by the composition and organization of ECM components. The anatomical variability in collagen, elastic fibers, and GAGs highlights the need for region-specific approaches in dermatological research and anti-aging treatments. Our study provides insight into the ECM composition of the FH and UE regions, highlighting significant differences in GAG distribution while showing relative stability in collagen and elastin. These findings suggest that GAG depletion may play a role in early skin aging, with distinct regional and orientation-based variations.

Within the FH, GAG content was significantly higher in horizontal sections compared to vertical sections, while collagen and elastin showed no significant differences. The stability of collagen and elastin in FH may be due to the FH’s thicker skin and higher mechanical load, which likely maintains a consistent ECM structure regardless of orientation. Unlike GAGs, which are more dynamic and hydration-dependent, collagen and elastin are structural proteins that degrade more gradually over time rather than redistributing across orientations. The higher GAG levels in horizontal sections may reflect the FH’s adaptation to tissue expansion and structural demands resulting from frequent facial expressions. Since GAGs are crucial for hydration and elasticity, their preferential distribution in horizontal orientations may help maintain structural integrity and flexibility in response to repetitive stress [12].

Interestingly, a similar pattern was observed in the UE region, where horizontal sections contained more GAGs than vertical sections, while collagen and elastin remained unchanged between orientations. However, unlike the FH, the UE region is intrinsically more delicate, with thinner skin and lower mechanical support. The lower GAG content in vertical sections may contribute to early structural weakness, reduced hydration, and volume loss, which are common age-related changes in this region. Given that the UE is one of the first areas to show visible signs of aging, the reduced GAG levels in certain orientations may indicate a higher susceptibility to dehydration and fine-line formation due to insufficient ECM support.

While orientation-dependent GAG variations were present in both regions, the FH exhibited a significantly higher amount of collagen and GAGs in vertical sections compared to the UE. This suggests that regional mechanical properties and structural demands influence ECM composition. The FH, being a thicker and more mechanically active area, may retain more collagen and GAGs in vertical orientations to withstand repeated stress from muscle contractions and facial movement. In contrast, the UE region, which lacks robust structural support, appears to have a lower baseline ECM content in vertical sections, making it more prone to early aging-related changes such as sagging and loss of elasticity.

Interestingly, the absence of significant differences in collagen and elastin levels between the horizontal regions of the FH and UE suggests that early facial aging may be linked to hydration-related changes. While collagen is often considered the hallmark of skin aging, its relative stability across regions and orientations in this study suggests that GAG depletion may be an earlier and more significant contributor to the initial stages of ECM deterioration. According to an early study, a change in the number of GAGs may be used to predict a change in the amount or content of water during the aging process [13]. This finding highlights the potential role of GAGs in maintaining skin hydration and structural integrity, suggesting that interventions targeting GAG preservation could play a crucial role in delaying the visible signs of early facial aging.

Moreover, aging-related changes in the ECM were significant, demonstrating the gradual decline in skin structure and function that naturally occurs over time. As the skin ages, the ECM’s composition and organization shift, resulting in a loss of elasticity, reduced hydration, and weakened structural integrity. These alterations contribute to the characteristic features of aging skin, including wrinkling, thinning, and diminished resilience. The consistency of these changes across male and female samples in this study suggests that intrinsic aging processes occur similarly regardless of gender. However, external factors like hormonal fluctuations, UV exposure, and lifestyle habits may still influence ECM remodeling in living individuals. Future studies with age-stratified samples and longitudinal analysis could provide more insight into the progressive nature of ECM changes over time.

Although our study offers important new information about the orientation-dependent and regional variations in the ECM composition of the FH and UE areas, there are several important limitations to consider. The findings may not be as broadly applicable to larger populations due to a smaller sample size. A more thorough knowledge of ECM variability would be possible with a larger sample size, especially when comparing various age groups, skin types, and demographic backgrounds. Even though cadaveric skin samples are useful for histological research, they fall short of capturing the dynamic processes of ECM remodeling that take place in living tissue. The absence of vascular supply, cellular turnover, and external factors like UV exposure and mechanical stress in these samples could have affected the observed outcomes. Future research with larger, more diverse samples and longitudinal, in vivo analyses is needed to better understand the complex interplay of structural and biochemical changes in facial skin aging.

The results of this study may have various implications for cosmetic medicine and dermatology. Since GAGs are lost in vertical UE portions, early hydration-focused interventions, including topical treatments or fillers based on hyaluronic acid, may help preserve the integrity of the ECM before the breakdown of collagen and elastin becomes noticeable. Moreover, the orientation-dependent variations in ECM distribution highlight the necessity of more accurate methods in cosmetic operations, such as filler insertion techniques that correspond with patterns of mechanical stress and natural hydration. These differences highlight how crucial it is to consider Langer’s lines when performing cosmetic surgery. Additionally, the relatively higher GAG levels in the UE horizontal sections suggest that this region retains some hydration potential despite its structural fragility. It has been shown that the pattern of GAG deposition in the dermis is critical, as sun-damaged skin accumulates GAGs on degraded elastic fibers rather than between collagen and elastin fibers, potentially disrupting collagen organization and dermal structure [14]. One would expect increased GAG content to result in youthful, supple skin; however, a study conducted to analyze GAG content in photoaged skin showed that the abnormal placement of GAGs may explain the paradoxical weathered appearance of photodamaged skin [15]. Clinically, this indicates that hydration-based treatments could be particularly effective when applied early, taking advantage of the existing ECM framework to support skin elasticity and delay visible signs of aging. Tailoring interventions to these regional differences may improve outcomes, especially in preventing premature volume loss and laxity in the UE area.

## Conclusions

This study provides insight into regional and orientation-dependent differences in ECM composition within the FH and UE areas. These findings contribute to our understanding of how ECM components vary across facial regions, with potential implications for dermatology, cosmetic medicine, and aging research. However, these results represent a preliminary step in understanding the complex relationship between ECM structure and facial skin characteristics.

Future research with larger sample sizes and diverse populations is essential to validate these findings. Studies that account for variables such as age, gender, environmental exposures, and anatomical differences across various facial regions could provide a more comprehensive picture of ECM dynamics. Additionally, investigating the molecular mechanisms underlying these regional differences may offer further insights into the aging process and inform strategies for more targeted and effective therapeutic interventions.
